# Challenges for nurses who work in community mental health centres in the West Bank, Palestine

**DOI:** 10.1186/s13033-016-0112-4

**Published:** 2017-01-03

**Authors:** Mohammad Marie, Ben Hannigan, Aled Jones

**Affiliations:** 1 College of Medicine and Health Sciences, Annajah National University, Annajah National University Hospital, West Bank, Palestine; 2 School of Healthcare Sciences, College of Biomedical and Life Sciences, Cardiff University, Eastgate House, 35-43 Newport Road, Cardiff, CF24 0AB UK

**Keywords:** Conflict zone, Mental health system, Challenges, Community mental health nurses, Palestine, West Bank

## Abstract

**Background:**

Nurses in Palestine (occupied Palestinian territory) work in a significantly challenging environment. The mental health care system is underdeveloped and under-resourced. For example, the total number of nurses who work in community mental health centres in the West Bank is seventeen, clearly insufficient in a total population of approximately three million. This research explored daily challenges that Palestinian community mental health nurses (CMHNs) face within and outside their demanding workplaces.

**Methods:**

An interpretive qualitative design was chosen. Face-to-face interviews were completed with fifteen participants. Thirty-two hours of observations of the day-to-day working environment and workplace routines were conducted in two communities’ mental health centres. Written documents relating to practical job-related policies were also collected from various workplaces. Thematic analysis was used across all data sources resulting in four main themes, which describe the challenges faced by CMHNs.

**Results and conclusion:**

These themes consist of the context of unrest, stigma, lack of resources, and organisational or mental health system challenges. The study concludes with a better understanding of challenges in nursing which draws on wider cultural contexts and resilience. The outcomes from this study can be used to decrease the challenges for health professionals and enhance the mental health care system in Palestine.

## Background

People in Palestine (occupied Palestinian territory) face severe challenges due to long-term political unrest and occupation. According to Marie et al. [1] civilians are waiting for an end to the occupation, to have freedom of movement and to have their basic human needs met. As a result of increasing numbers of people being in need of mental health care [1] there is a necessity to increase resources and improve the availability and quality of services in order to better meet these. The whole health workforce faces chronic difficulties in managing daily life inside and outside the workplace [1]. These difficulties need to be understood and discussed in the context of the collective suffering of the Palestinian population. There is also a need to enhance the resilience and capacity of community mental health teams [2].

The health care system is fragmented and complex due to a long history of occupation and conflict. Basic public health and primary care (including mental health services) are offered by four main facilities: the Palestinian Authority (Governmental), the United Nations (United Nation Relief and Work Agency for Palestinians), non-governmental organizations (NGOs), and private health care services such as psychiatric clinics. Most of these services are supported by international donors and the health budget is influenced by the political situation [3, 4]. According to the World Health Organization (WHO) there is a lack of human and other resources in the health sector in Palestine. For example, the ratio of primary care physicians is 17.2 per 100,000 [3]. There are two main psychiatric hospitals (one in each territory, Gaza and West Bank), and 42 private and public outpatient mental health facilities in Palestine [3]. There is also a severe lack of mental health professionals who work in the field. The total number of people working in private practices or mental health facilities is 268 (7.3 per 100,000 population) [3]. This number includes 125 nurses (3.34 per 100,000 population) and 32 psychiatrists (0.87 per 100,000 population) [3]. Nurses who work in outpatient facilities are only 27 in West Bank and Gaza; 17 of them work in the West Bank [3]. These nurses offer mental health care to a population of around three million civilians [5]. This is considered very low in comparison with other countries such as the United Kingdom, where there are more than 900 community mental health nurses (CMHNs) in Wales who offer services to a similar sized population [6].

Health workers face difficulties accessing their workplaces due to movement restrictions [7]. Palestinian nurses work within an environment which lacks safety and face personal threats [8]. They need help and support in their workplace [9] as they face high levels of stress as a result of multifaceted occupation-related difficulties and increasing home and family responsibilities [10]. For example, midwives, as well as nurses, face similar challenges due to the Separation Wall and movement restrictions [11].

One of the major challenges faced in Palestine is that health services managed by the Palestinian Authority have struggled with financial challenges since their creation in 1995. The financial situation has been affected by sanctions imposed by Israel, and the lack of international funds [12]. The lack of resources is a challenge experienced by all nurses in Palestine [11]. Nurses and midwives receive inconsistent salaries, work long hours, suffer from severe staff shortages, and have excessive workloads. Hassan and Wick [10] found that nurses and midwives work in poor work environments and receive little motivation. They are faced with a lack of medical supplies and equipment and also the absence of regular monthly financial payments. This lack of resources can be related to the context of political restrictions before and after the management of the Palestinian Authority. The lack of an effective nursing union to lobby for more resources is also a factor [10].

The financial challenges are particularly relevant to mental health services which provide care in one of the areas of clinical practice with the greatest health need in Palestine [13]. According to the WHO and the Palestinian Ministry of Health (MOH) [3] mental health services have been the most affected by the chronic lack of funding. Mental health nurses have been unable to offer the level of care they wish to, with differences emerging between what they have learned and what they are able to do in reality faced with excessive workloads and a lack of resources [14]. Even before the Palestinian Authority assumed responsibility for the healthcare system in 1995, Manasra [15] observed that nurses, working in the mental health field, experienced low wages and severe staff shortages. There were also little or no opportunities to carry out home visits or opportunities for continuing education or professional development.

As well as financial and resource difficulties other challenges exist within the workplace. According to the Coalition for Accountability and Integrity (AMAN) [16], favouritism is one of the major types of corruption in the health sector in Palestine. According to Wick [11] and Hassan and Wick [10] Palestinian nurses and midwives have a low status in comparison with physicians who manage national health policies in the main, and nurses and midwives have limited opportunities to advance. A further challenge is that, according to McAuley et al. [14], local society creates challenges for nurses due to a lack of awareness of, and stigma towards, mental illness. Furthermore, some nurses have been keen to practise new strategies which they have learned, but some of their colleagues in mental health teams have been resistant to change. For instance, physicians have often refused to involve nurses in assessment, evaluation and treatment plans effectively. McAuley et al. [14] also found that some psychiatrists described nurses as receptionists or clerks, with nurses having a status considerably lower than that of physicians, psychologists and social workers. There is no evidence that CMHNs receive clinical supervision or use reflective practices. For example, according to Manasra [15] mental health nursing is described as not being a desired job due to the associated stigma.

## Aims

Against this background, the aim of this paper is to observe and describe the environment within community mental health workplaces, and to explore the challenges facing Palestinian CMHNs.

## Methods

An interpretive qualitative design was chosen. Data were generated from all mental health settings in the West Bank which were 12 outpatient facilities. These included all the directorates or cities in the West Bank: Hebron, Halhul, Bethlehem, Jericho, Ramallah, Nablus, Salphit, Qalqelia, Jenin, East Jerusalem, Tulkarim and Azoon town beside Qalqelia city. In each site, there are only one or two nurses who work in community mental health centres at the most. Eleven outpatient facilities were governmental and one centre belonged to the NGO. Other settings were excluded due to the impossibility of access because of the lack of freedom to travel to places such as Jerusalem and the Gaza strip. An attempt was made to interview the total population of CMHNs known to be working in the West Bank (n = 17), of whom 15 agreed to participate. Fourteen nurses worked in the governmental outpatient facilities and only one nurse worked in the NGO. The sample consisted of seven male and eight female nurses. Thirteen were married; one of the females was divorced with one daughter and one of them was single. The ages of the participants were between 24 and 60 years old. They had qualifications in general nursing from diploma to bachelor’s degree level except one who had a secondary school degree in nursing. One of the nurses had a master’s degree outside the health field but none of the sample had a master’s degree in nursing or mental health nursing. However, the interviewees had received brief lessons in the mental health field during the undergraduate period or had undergone short training courses after their graduation. These nurses who worked in the outpatient facilities were treated or called by their employer (the Ministry of Health) ''community mental health nurses''. Interviews were conducted by the first author in Arabic, and lasted from 30 to 90 min. Interviews addressed the following main items:

1. Tell me about your work challenges.
2. Tell me about the resources supporting you to cope with work adversities.
3. Tell me about your life challenges.
4. Tell me about the resources enabling you to keep going.

Multiple documents (such as operational policies) from the 12 sites were collected [17]. Purposive sampling was used in the selection of sites in which to generate observational data. Observations of the day-to-day working environment were conducted in one of the Governmental community mental health centres and in a non-governmental organisation (NGO) centre. The lead author (MM) spent a maximum of 4 hours over each of eight days observing in both centres. The choice to conduct fieldwork in two separate, institutionally distinct, workplaces was made to enable comparison and contrast [18].

Ethical approval from the Research Ethics Committee (REC) in what was then the School of Nursing and Midwifery Studies at Cardiff University was secured in 2012. Data were generated from June until the end of that year, following the securing of official permission from the Palestinian Ministry of Health. Before participation in the study, staff and service users were informed of the project plans and individuals were offered the chance to discuss the purpose of fieldwork. Signed informed consent was obtained from all staff before their interview participation. Pseudonyms were used to protect the identities of interviewees, people observed in the work settings and named in collected documents. Audio-recorded interviews were saved in password-protected computers and other collected resources were kept in a locked office drawer.

Braun and Clarke’s [19] method was used to thematically analyse the data. The complete dataset included field notes from observations, collected written documents and transcribed interviews with nurses. Data were analysed to answer the research objectives related to the challenges faced by nurses. Reliability and validity were taken into consideration to increase the quality of this research [20]. Two audio recorders were used to record all interviews. Two native Palestinians carried out transcriptions in the colloquial language (local Palestinian dialect), and both made comparisons between the two recorders to confirm accuracy and reliability [21]. As a native Arabic speaking researcher, MM conducted the interviews in the same language as the participants with local Palestinian dialect (urban and rural). The data was analysed in the same language, with quotes translated into English by an official translator. These were then reviewed to ensure the accuracy of the translation. More than one coder can carry out analysis to promote rigorous interpretation [20], and here five people shared their insights during the analytic process [22] using the same thematic approach [20].

The first author and the first independent co-coder, who is Palestinian, completed coding for all the generated data (interviews, field notes and the collected documents). To promote rigour two interviews were translated into English in their totality, and were co-coded by a native English speaker. Both co-coders have backgrounds outside the nursing field and each carried out their work separately. Co-coders had no contact with each other during the data analysis process. All used the same method of analysis which is based on induction to ensure reliability. After finishing coding, the three met to discuss and agree themes. Field notes were written in English hour by hour. Field notes were available to all authors of this article, along with the two translated interviews. All authors discussed the method of analysis and shared insights, this whole process being designed to enhance objectivity and minimise subjectivity and bias [19].

## Results

Four main themes are presented that describe the challenges faced by CMHNs. These themes consist of the context of turmoil and unrest challenges; stigma; lack of resources; and organisational challenges.

## The context of turmoil and unrest

### Lack of safety and freedom

Participants experienced feelings of lack of safety and a sense of injustice inside and outside the health workplaces. In the extract below with one of the interviewees, a nurse, talked about a wide range of challenges including financial, economic, transport and the effect of the Separation Wall in his village:

*M: Financial challenge, it’s the first and the difficult one, followed by living and social challenges…*

*MM: Can you discuss other challenges?*

*M: Transportation.*

*MM: What about transportation?*

*M: Every morning I have to walk a long distance; about 15 to 30* *min or one km, because my house is far from the village and cars are not allowed to come there. Moreover, the Apartheid Wall forms a basic barrier in our life; it changed our life upside down.*

*MM: How has it changed your life?*

*M: My village was economically prosperous. People worked 24/7, but now my village has become isolated; there is neither transportation nor commercial movement*–*nothing*– *and this has an effect on the current conditions.*

*[M.A. Interview, Nongovernmental Organisation (NGO) nurse]*

The above extract demonstrates how nurses have to work within a zone of political conflict which is demanding and sometimes overwhelming. Participants experienced lack of freedom to travel, and they talked about having been exposed to risks while travelling on the roads. For example, nearly all of the nurses reported experiences when Israeli forces or settlers threatened them personally. The movement restrictions limited the travelling of nurses between districts to workplaces, to conduct meetings or attend workshops. One of the nurses used alternative ways to come to his workplaces when he was prohibited from passing the military barrier:

*T: Sometimes, I used to come to my job through one of the sewage canals there!*

*[T.H. Interview, Governmental centre]*

Nurses described violations to their basic human rights especially when they went to work during times of curfew or clashes:

*S: during that period of time, we sometimes ran into soldiers who threw gas canisters at us. We had to run and run. The distance was too long. We rarely found a car to take us. I remember that I had walked 20* *km on my feet many times.*

*[S.S. Interview, Governmental centre]*

They also experienced a lack of freedom to speak about or show their political interest within a politically fragile environment. CMHNs were concerned by unrest in the whole of their lives not just while on duty:

*F: You know I was discharged from my job because of my husband’s political activity… You definitely know about the military barriers and the difficulties that we face if we decide to go to Jerusalem, for example. Also the economic conditions and the high costs of living; we have to pre*-*set our budget before deciding to buy anything…Similarly, if I went to work, and left my son sick at home, or if my daughter or son had exams, I will be worried all the time until I call them and ask how they did, whether they have eaten or not, whether they have arrived home safely…*

*[F.N. interview, Governmental centre]*

## Lack of support

Participants reported that there was a low level of formal support for nursing and nursing development, especially from their employers and nursing association. In the following extract, a nurse expressed her feelings when we talked about the available supportive facilities or rights for nurses. She was talking about nurses being marginalised by the Ministry of Health:

*M: What else does marginalisation of nursing mean? How do you feel that you are marginalised here at the Centre?*

*S: Nobody here supports us. There are things that I do not know enough about in nursing.*

*M: In what aspects do you need support?*

*S: For example, the Ministry should send us to attend courses so that we could know more…Here at all of our ministries, nobody supports or motivates us.*

*[S.S. interview, Governmental centre]*

In the next extract, a participant was discussing the available support and respect shown to nurses. The nurse expressed her dissatisfaction with the level of respect or available opportunities to be listened to, and talked about the frustrating environment inside the Ministry of Health:

*M: Do you feel that opportunities are available for you here as a nurse?*

*L: No, no, no. Not even one in a thousand, neither financial, nor the chance to express ourselves, we are always frustrated…*

*[L.E.J. Interview, Governmental centre]*

Nurses thus felt a lack of formal support within the context of turmoil and unrest. This challenge included lack of support from their employers, the nursing union, and other mental health institutions.

## Inconsistency of care services delivery

There was inconsistency of care services due to lack of health care supplies and instability within health care services. The extended field note below goes on to provide an example of the lack of basic medical supplies and medications experienced in one of the governmental health centres:

*I observed in the second governmental centre that the nurse (TH) was unable to give the intramuscular injections to the patients in the mental health centre; I asked the nurse (TH), ‘why’? He replied:’we (mental health team) have had no syringes and needles in our centre for two months’.*

*[Field notes, Governmental centre]*

The inconsistency of delivering care services was because of limited financial abilities of the Palestinian Authority. The chronic economic hardship of the local authorities limited the expenses available to pay the nurses and meet the needs of service users, with significant consequences as revealed in this field note extract:

*During the observation period the Governmental Mental Health Centre closed due to the employees’ strikes. The governmental health employees had not received regular salaries for months. This meant that the availability of medications was sporadic and this might cause relapses in the patients.*

*[Field notes, Governmental centre]*

The data led us to conclude that the health system was significantly affected by the financial crisis. The availability and delivery of care services such as medication was also affected by practical constraints imposed as part of the conflict with Israel.

## Stigma toward mental illness challenge

The data generated demonstrated a significant lack of public and health professionals’ awareness of, plus considerable social stigma towards, mental illness. This was partly due to a lack of ‘raising awareness’ programmes as a result of lack of funding. For example, one nurse reported that she was working in the governmental centre and explained how she had encountered stigma:

F: When I was employed here **** (name of the centre) they, including nurses in the primary health department, ridiculed and laughed at me, they said that I am crazy to work here in the clinic. For your information, when this job was offered to several nurses no one accepted it but me…We face difficulties when we try to integrate mentally ill people in society, and to remove barriers between them and the normal people. Our patients have creative abilities when they get the opportunity to work and innovate. However, when they are treated as mad, their condition will worsen.

*[F.N. interview, Governmental centre]*

The NGO nurse also reported that many people were afraid to join the programme in the centre. Families and clients refused to join in the main because of the social stigmas of mental diseases. Their neighbours might learn that the family has a mentally ill member. Some of the people did not value the importance of the centre’s activities.

## Lack of resources

### Lack of funding

There was a lack of human resources, learning opportunities, and supervision or guidance, plus poor facilities and infrastructure and a lack of financial resources. Only one or two nurses at the most covered each district, which might consist of a population numbering a quarter or half a million. There was rarely any replacement of employees who retired or took leave [2].

In addition to the issue of accessing staff development and training already touched upon earlier in this paper, participants took the opportunity to further describe a near absence of teaching, effective guidance or supervision, and very limited up-to-date training to manage the challenging psychiatric symptoms experienced by people on their caseloads:

*F: I could not pay visits to patients at home because there is not another nurse to cover me. Similarly, I could not engage in any activity within the centre… Definitely. I need someone to guide me in my working; to tell me whether my performance is good or not, whether I do certain things correctly or not. For example, if I have someone to train and teach me new methods to deal with the mentally ill, and correct my mistakes, then my skills and knowledge will be improved, and I will base my work on scientific grounds when dealing with patients; whereas if I work alone without supervision or learning new tactics, I will never enhance my skills and they will stay dead.*

*[F.N. interview, Governmental centre]*

There was a low status of “mental health” generally and towards nursing work. Nurses had very limited financial rewards (wages) and motivators, as this participant described:

*S: Now I attend work at the health centre but the salary*—*there are no rewards, no bonuses*—*no motivators*—*you work hard, but you do not receive even a thankful word, even if you work more than your capacity. When I worked at the Central Health Department, I used to go out on vaccination campaigns and other kinds of campaigns and visits and so on, but you would not receive a thankful word. Nevertheless, if you make a mistake or an error, the world will turn upside down. This means that your work is not appreciated, no motivators, nobody appreciates your work. Frankly, neither the place, nor the team, nor the management motivates you. There are neither financial nor moral motivations.*

*[S.S. interview, Governmental centre]*

These shortages of resources were associated with the military/political conflict and a lack of funding from the Ministry of Health. Some participants also reported that the nursing association did not take any serious action to lobby for more resources.

## Managing psychiatric symptoms

Nurses described challenges related to managing the mental health difficulties experienced by service users. One participant confirmed the problem of recurrent relapses of patients:

M: Patients’ numbers declined; those who were coming from **** (city) stopped because of the long distance and lack of transportation. Therefore, we try to bring them in. As regards the patients from ***** (city) and its villages, we offered the transport but at their expense. Some of them were able to stay with the programme, others were not. When they relapse, they ask for help from us.

*[M.A. Interview, NGO nurse]*

As this analysis reveals, the lack of resources, including the inconsistency of care services, lack of medication, lack of teaching, and lack of commitment or capacity of service users to fulfil their treatment plans increased the problem of relapses. Service users stopped taking medication when they were unable to manage side effects, leading to relapses. This occurred due to lack of teaching for families and patients in the context of medication management. Lack of home visits by CMHNs to follow up care at home increased the risk of relapse and deterioration of mental illness. The lack of home visits occurred due to severe shortages of staff and misconceptions about nurses’ roles. The nurses had to manage with a lack of supportive facilities.

## Organisational challenges

### Gaps between theory and practice

There was a gap between what nurses had been taught in textbooks and what they were able to offer in practice. In the extended extract below a participant explained that he is unable to provide what he has been taught to offer:

*T: You should not give the patient medication and ask him to leave. You should have enough time for each patient to take care of him. You should have a private room so that the patient can come and you should know how he takes the medication, what side effects are there…This is how nursing should be, but nursing is something and the reality is another.*

*[T.H. interview, Governmental centre]*

There was therefore a gap between the reality of the mental health system and what nurses learned in the schools or universities.

### Professional status of nursing

There were challenges related to the professional status of nursing, as one participant explained:

*F: Here in the department, (I hope no one hears) the mental health nurse is completely ignored; they rarely engage us in workshops, courses or any other activity they do…Consequently, you feel there is no importance of your role.*

*[F.N. interview, Governmental centre]*

In the following interview related to transition from clinic to centre, one of the female nurses reported that she needs to feel involved and share effectively in development plans for the mental health care system:

*F: As I told you, I am ready to fight hard for anything related to mental health nursing. In fact, the mental health nurses are entirely neglected, the Ministry of Health has never nominated us in meetings or on courses or any other activity. I have been working here more than three years, and they never invited me to participate in a meeting or discussion.*

*[F.N. interview, Governmental centre]*

CMHNs therefore felt disempowered and marginalised, experiencing a lack of professional identity and autonomy. The low status of nursing can be linked with the bias and favouritism inside and outside the workplaces caused by the attitude of the local community. There was an unfair distribution of the limited training opportunities due to partiality. In the following extract, one of the nurses narrated her story when the Ministry of Health sent a psychologist to attend training for the mental health nurses instead of sending her. The psychologist has a close relative working in one of the leading positions in the Ministry of Health:

*F: …As I told you, when the psychologist went to Lebanon in order to participate in the training course, she was surprised that this course was originally made for the mental health nurses. Consequently, they changed the programme because she is a psychologist. And for your information, they were taught and trained by a mental health nurse in Beirut.*

*[F.N. interview, Governmental centre]*

Most of the nurses had not had development opportunities for many years. They reported that they need to do special training to fulfil their roles as mental health nurses. One of the nurses talked about her feelings toward status and respect:

*M: Do you feel that there is respect for nursing?*

*L: Rarely, perhaps in some departments. But here the physicians, especially the older ones, deal with us by looking down on us. I do not know why.*

*[L.E.J. Interview, Governmental centre]*

The entire sample expressed their significant dissatisfaction with the low status of the nursing profession within the Ministry of Health. One of the nurses stated during the interview when we discussed how some of the managers treat nurses:

*S: The management style is very bad. They deal with us in a dictatorial way…*

*[S.S. interview, Governmental centre]*

There was bias and favouritism against nurses or mental health nurses in particular. This resulted in CMHNs being disempowered, feeling marginalised, experiencing a lack of professional identity and autonomy. These challenges were mostly linked with lack of support and resources. This challenge is also possibly associated with the stigma toward the “mental health system” generally and the low status of nursing inside and outside the health organisation.

### Inter-professional challenges

Challenges here included lack of clarity over work roles. For example, one nurse said:

*Ab: I carry out tasks in addition to my duties. For example, I am here a nurse, a registrar, a pharmacist, offer care and vaccinations, and all other kinds of work. I also clean the clinic.*

*[Ab. interview, Governmental centre]*

Challenges therefore included a lack of clear job descriptions, multiple responsibilities and role overlaps, plus a lack of coordination and communication:

*There was a lack of communication or coordination with other community mental health centres in other cities.*

*[F.N. interview, Governmental centre]*

As this analysis reveals, organisational features including theory/practice gaps, low status and lack of role clarity were major challenges for nurses within the health system.

## Discussion

This wider context of turmoil and unrest creates challenges in work and in life in general which CMHNs in Palestine have to face. This resonates with findings found in studies conducted elsewhere in the world. Other scholars have discussed lack of safety and freedom among nurses in their workplaces during times of conflict. According to Birchenall [23] British nurses in the Channel Islands experienced challenges such as lack of, or inconsistency in, medical supplies, foods, and fuel during World War and German occupation. They worked in a violent environment while oppression and curfews were common. They carried out their nursing care in spite of surrounding challenges, such as some of the nurses being arrested or killed.

The theme of societal challenges consists of stigma toward mental illness. This study produced findings which can be linked to findings from previous work outside Palestine. For example, according to a WHO report [24] stigma toward mental illness is considered one of the most significant challenges in low and middle-income countries. There is a lack of funding and resources, which deters nurses from remaining in or joining the mental health field. According to Okasha et al. [25], there is misunderstanding of mental illness in the Arabic cultural context. According to Gazzaz [26], nurses face societal challenges in Saudi Arabian culture due to tradition and tribal society. Nursing is an underprivileged career and is attached with negative stereotypes in Saudi society. Lovering [27] also argues that nursing has a low status, especially amongst nurses, in Middle Eastern societies.

The challenge of a lack of resources can be linked to findings from previous work outside Palestine. Jenkins et al. [28] found UK mental health nurses experience a lack of human resources and face high workloads. Edwards et al. [6] found a lack of resources contributes to stress amongst CMHNs in Wales, UK whilst in a study of community psychiatric nurses (CPNs) in the UK Hopkinson et al. [29] found that staff face many resources challenges. CMHNs also need to expect relapses of service users, especially those who have psychosis [30]. According to Birchenall [23], the British nurses under German occupation during 1940–1945 also reported that they worked within an environment lacking training and resources. Finally, according to Okasha et al. [25] there are a lack of support and a lack of human resources including nurses, in the Arabic region.

Other scholars have discussed the organisational challenges facing CMHNs in their workplaces. Coffey and Hannigan [31] show how CMHNs in England and Wales face professional and inter-professional challenges. Hopkinson et al. [29] also found that CMHNs face challenges such as low status, lack of control, limited autonomy, lack of clear job descriptions, and roles which overlap. Murphy [32] reported that CMHNs are sometimes exposed to violence from service users within their workplaces, whilst Coffey and Hewitt [33] reported that CMHNs face challenges related to the mental health difficulties experienced by people using services. Nurses elsewhere in the world are working in multifaceted mental health systems which contain difficult challenges [34], whilst Edwards et al. [6] found how lack of support contributes to stress among CMHNs in Wales, UK. These findings link with the organisational challenges that Palestinian CMHNs face. Gaps between theory and practice identified in this current study have the additional potential to negatively affect the quality of health services and nurses’ morale and functioning.

## Recommendations and limitations

This study is significant for two main reasons. First, it is an example of qualitative research in a part of the world where qualitative studies are few. Second, it is research investigating daily challenges and problems facing nurses, particularly those who are working in the mental health field in Palestine. Again, this kind of research is exceptionally rare. However, the study also has a number of limitations. It was limited by time, funds and by the inclusion of Palestinians who lived in the West Bank only.

Study findings support a number of recommendations and implications for nursing practice. These courses of action should decrease stigma, and raise awareness toward mental illness inside and outside the workplace.

1. Challenges for Palestinian nurses must be decreased as much as possible, and there are a number of important changes which can be applied. For example, actions should be taken to support nursing associations, to enhance their status.
2. Managers and policy makers need to evolve strategies to enhance transparency and prevent favouritism and nepotism in the health system. More action should be taken to distribute available resources based on performance and achievement not on political and social affiliation. There is a necessity also to enable nurses to be given their rights in an official way and improve the sense of justice inside their workplaces.
3. There is a requirement to increase availability of resources among nurses. There is a need to increase the efficacy of care services by increasing medical supplies and nursing equipment. It is also essential to increase the budget of mental health services, so that they receive a realistic percentage of the Ministry of Health budget. The Ministry of Health and government must also increase the very small number of CMHNs, in order to enable them to apply high quality health care effectively and in the manner they would wish. For example, the CMHNs who have recently completed master’s degrees in community mental health nursing should be offered employment immediately, so they can start working in the community mental health centres.
4. There is a need to increase the continuous and up to date educational opportunities for current nurses. One strong recommendation is for nurses to share learning and support with each other through involvement in workshops, meetings, conferences, and in the transformation process.
5. Another important practical implication is to decrease the suffering of the nurses by improving the status of nurses and increasing their feelings of self-respect, autonomy, and identity. There is a need to produce clear job descriptions and decrease their excessive workloads and multiple responsibilities. It is essential to appreciate and value the performance of nurses within the health system. There is a definite need to listen to the health workers’ needs and suggestions carefully in order to solve problems associated with health and nursing care.
6. Nurses need to be involved effectively in managing available resources in order to enhance the quality of health care. For example, more actions should be taken to involve nurses in designing and implementing the strategic plans of, and health policies within, the mental health system. The leading positions inside the Ministry of Health need to include nurses; for example the Minister of Health or one of his assistants. Managers and policy makers need to enable nurses to lead and offer them the opportunity to be innovative in developing and applying better health care. Taken together, these findings recommend the need to increase the control nurses have in managing available resources. This recognition would serve to increase motivation, a sense of reward among nurses and improve quality of health care.
7. The findings of this research also support the idea of developing nurses’ resilience. This means that nurses can learn how to be resilient through training and learning from experiences in work through reflection and supervision. Developing resilience can also be through supporting the idea of educating nurses how to enhance their coping skills and develop endurance, self-confidence and efficacy. These strategies can be consistent with or inspired by their Sumud and Islamic cultures [2], helping CMHNs find meaning in their suffering as part of the Palestinian people.

## Conclusion

**Fig. 1:**
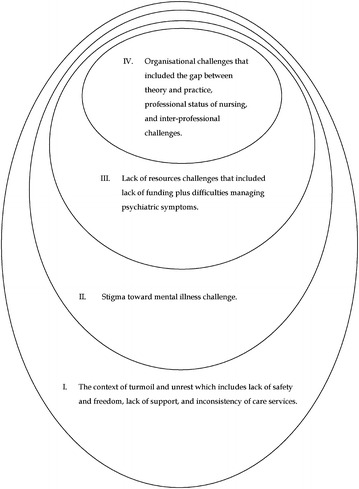
Challenges to the nurses who work in community mental health centres in Palestine

Figure 1 below summarises the challenges to CMHNs, showing how these were embedded within the health system and a wider context of turmoil and unrest. For instance, lack of freedom or occupation practices can create challenges for nurses such as lack of funding which can lead to lack of resources. Lack of resources can cause difficulties in managing psychiatric symptoms or increase the gap between theory and practice. There is also a lack of programmes to increase awareness toward mental illness which can be associated with the lack of financial capability within the Palestinian Authority.

The significance of the findings in this study is that the challenges faced by nurses in Palestine are of a much greater magnitude than those found in many other parts of the world. These challenges are embedded within a wider context of occupation, turmoil and unrest. Shortages of rewards were associated with the military/political conflict and a lack of funding from employers, and with the lack of an effective parliament to determine or protect the rights of the employees. Challenges for nurses were also associated with the lowly status of mental health generally, a bias against nursing work and a lack of an effective nursing association to lobby for rewards, bonuses, and motivators.
